# Resting Tension Affects eNOS Activity in a Calcium-Dependent Way in Airways

**DOI:** 10.1155/2007/24174

**Published:** 2007-03-28

**Authors:** Eudoxia Kitsiopoulou, Apostolia A. Hatziefthimiou, Konstantinos I. Gourgoulianis, Paschalis-Adam Molyvdas

**Affiliations:** ^1^Department of Physiology, Medical School, University of Thessaly, Papakiriazi 22, 41222 Larissa, Greece; ^2^Department of Respiratory Medicine, Medical School, University of Thessaly, Papakiriazi 22, 41222 Larissa, Greece

## Abstract

The alteration of resting tension (RT) from 0.5 g to 2.5 g increased significantly airway smooth muscle contractions induced by acetylcholine (ACh) in rabbit trachea. The decrease in extracellular calcium concentration [Ca^2+^]_o_ from 2 mM to 0.2 mM reduced ACh-induced contractions only at 2.5 g RT with no effect at 0.5 g RT. The nonselective inhibitor of nitric oxide synthase (NOS), N^G^-nitro-L-arginine methyl ester (L-NAME) increased ACh-induced contractions at 2.5 g RT. The inhibitor of inducible NOS, S-methylsothiourea or neuronal NOS, 7-nitroindazole had no effect. At 2.5 g RT, the reduction of [Ca^2+^]_o_ from 2 mM to 0.2 mM abolished the effect of L-NAME on ACh-induced contractions. The NO precursor L-arginine or the tyrosine kinase inhibitors erbstatin A and genistein had no effect on ACh-induced contractions obtained at 2.5 g RT. Our results suggest that in airways, RT affects ACh-induced contractions by modulating the activity of epithelial NOS in a calcium-dependent, tyrosine-phosphorylation-independent way.

## 1. INTRODUCTION

Nitric oxide (NO) is released by a wide variety of cell types including epithelial cells, nerve, and inflammatory cells in airways [1]. NO is the end product of the conversion of L-arginine to L-citrulline and this reaction is catalyzed by NO synthase (NOS). Functionally, NOS isoforms are distinguished into a constitutive (cNOS) form and an inducible (iNOS) form [2]. The constitutive isoforms of NOS, neuronal (nNOS), and endothelial (eNOS) seem to protect airways from excessive bronchoconstriction, while iNOS has a modulatory role in inflammatory disorders of the airways such as asthma [3].

Constitutive NOS is activated by an increase in intracellular calcium concentration that in turn promotes calmodulin binding to NOS and releases low amounts of NO for short periods in response to receptor and physical stimulation [4]. Studies in vessels provide convincing experimental evidence that eNOS may be stimulated by two independent signaling pathways and is differentially activated by receptor-dependent agonists and mechanical stimuli. Particularly, the activation of eNOS by receptor-dependent agonists like acetylcholine, histamine or bradykinin is mediated by an increase in intracellular calcium [4], while its activation by mechanical stimuli like shear stress is induced by its phosphorylation [5–7].

In rabbit trachea, airway epithelium modulates the responsiveness of airway smooth muscle (ASM) to acetylcholine depending on the initial tension [8, 9]. This effect was shown to be mediated, at least in part, via NO release [9]. Therefore, the purpose of this study was to investigate the effect exerted by the resting tension (RT) of airways smooth muscle on activation of eNOS and the mechanism(s) involved.

## 2. METHODS

Contractility studies were performed with tracheal strips obtained from adult male or female rabbits (approximately 2 Kg body weight). Rabbits were maintained in individual cages under a controlled environment consisting of a 12-hour light-dark cycle and ambient temperature of 22°C, were provided with food and water before use for the study, and were treated in compliance with ethical and institutional guidelines. Animals were sacrificed by an overdose of intravenously administered sodium pentobarbital (Vétoquinol, France). Exothoracic tracheal tissue was removed and placed in Krebs solution (pH 7.4 at 37°C) with the following composition (in mM): Na^+^ 137; Mg^2+^ 1.1; K^+^ 5.9; Cl^−^ 123.0, Ca^2+^ 2, H_2_PO_4_^−^ 1.2; HCO_3_^−^ 24.9, and glucose 9.6. The solution was gassed with 95% O_2_ and 5% CO_2_. In experiments carried out in Krebs solution with low calcium concentration, the solution had the same composition except calcium concentration that was 0.2 mM. The extracellular calcium concentration 0.2 mM has been chosen because it is lower than the suggested calcium threshold for epithelial modulatory part on ACh-induced contraction [10] and did not affect ASM passive tension.

The trachea was cleaned of surrounding connective tissue and tracheal strips (2 mm wide, 14 mm length) were obtained from tracheal rings dissected from the middle trachea with the assistance of SZ30 Olympus stereoscope. The thickness of smooth muscle layer was measured with the assistance of an inverted microscope (DIAPHOT 300 Nikon), a color video camera (TK-1281, JVC) and monitor (TM-290ZE, JVC), as well as by using a caliper (0.0025 mm^2^ resolution). Then the cartilaginous rings were cut opposite to the smooth muscle layer. Each strip was placed with the superfused luminal side up in a water-jacketed organ bath. One end of the cartilage was used to pin the preparation to the Sylgard 184 (Dow Corning) bottom of the horizontal organ bath, whereas the other end was used to mount the strip to the force-displacement transducer. Tracheal strips were stretched manually to 0.5 g or 2.5 g RT and were allowed to equilibrate for at least 60 minutes. Preliminary experiments have shown that at 2.5 g RT, the developed tension of ASM to ACh lies within the linear part of the RT-tension curve.

The entire strip was continuously perfused with oxygenated Krebs solution at 37°C. Acetylcholine 10^−9^ M to 10^−3^ M was added cumulatively to the organ bath. Changes in tension were recorded on a Grass FT03C force-displacement transducer and were displayed via a Grass 7400 physiological recorder.

In experiments in which N^G^-nitro-L-arginine methyl ester (L-NAME, 10^−4^ M), S-methylisothiourea (SMT, 10^−4^ M), 7-Nitroindazole (7-NI, 10^−4^ M), L-arginine (10^−3^ M), erbstatin A (3 × 10^−6^ M), and genistein (3 × 10^−6^ M) were used, strips were incubated with each of the above agent for 30 minutes before acetylcholine was added.

The maximal active tension generated in response to different concentrations of acetylcholine was calculated; values are expressed as force in grams per cross-section in millimeters (g mm^−2^). All data are given as means ± standard error (SE) and *N* refers to the number of animals. The data were compared by one-way analysis of variance (ANOVA) with statistically significant differences between groups being determined by Bonferroni's post-hoc test, while statistical differences between two groups were done by Mann-Whitney independent samples test. A comparison is considered significant when *P* < .05. The statistical analysis was performed using SPSS v11. The curve fitting and graph drawing were carried out using the graphical package Sigma Plot 2001.

Acetylcholine, L-NAME, SMT, 7-NI, L-arginine and genistein were obtained from Sigma (Germany). Erbstatin A was obtained from Calbiochem (Calif, USA).

## 3. RESULTS

**Figure 1:**
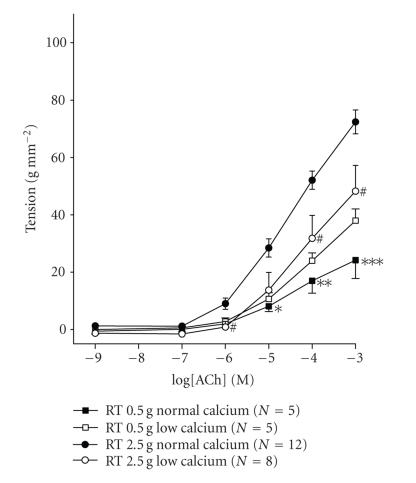
Concentration-effect curves for acetylcholine, performed in normal and low extracellular calcium concentration Krebs solution, at 0.5 g (squares) and 2.5 g (circles) RT. Data are means from 5 to 12 experiments and vertical lines show SE. # *P* < .05: comparison between values of tension at low and normal extracellular calcium concentrations, obtained at 2.5 RT. *P < .05, **P < .01, and ***P < .001: comparison between values of tension obtained at 0.5 g and 2.5 g RT at normal extracellular calcium concentration.

The alteration of RT from 0.5 g to 2.5 g increased significantly contractions induced by 10^−6^ M to 10^−3^ M ACh (*P* < .05) (Figure 1). This effect of RT on the responsiveness of ASM to ACh depends on extracellular calcium concentration. Thus, at low calcium concentration there was no difference in ACh-induced contractions obtained at 0.5 g and 2.5 g RT (Figure 1). The isometric forces developed by 10^−3^ M ACh were 24.18 ± 6.34 g mm^−2^ and 72.41 ± 4.15 g mm^−2^ at 0.5 g and 2.5 g RT, respectively (*P* < .001, Figure 1). At low extracellular Ca^2+^ concentration, the isometric forces developed by 10^−3^ M ACh were 37.97 ± 4.07 g mm^−2^ and 48.26 ± 8.95 g mm^−2^ at 0.5 g and 2.5 g RT, respectively (Figure 1). At 2.5 g RT, the decrease in extracellular calcium concentration reduced contractions induced by 10^−6^, 10^−4^, and 10^−3^ M ACh (*P* < .05), (Figure 1) with no effect on ACh-induced contractions obtained at 0.5 g RT.

**Figure 2:**
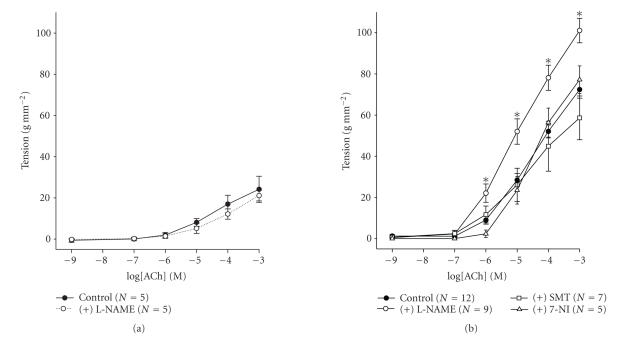
(a) Concentration-effect curves for acetylcholine at 0.5 g RT and normal extracellular calcium concentration, in the absence or in the presence of L-NAME. Data are means from 5 experiments and vertical lines show SE. (b) Concentration-effect curves for acetylcholine at 2.5 g RT and normal extracellular calcium concentration, in the absence or in the presence of the nonselective inhibitor of NOS, N^G^-nitro-L-arginine methyl ester (L-NAME), the inhibitor of iNOS, S-methylsothiourea (SMT), and nNOS, 7-Nitroindazole (7-NI). Data are means of 5 to 12 experiments and vertical lines show SE. *N* refers to the number of animals studied. *P < .05 compared to control values.

At 0.5 g RT, the presence of L-NAME, a nonselective NOS inhibitor, had no effect on ACh-induced contractions (Figure 2(a)). On the contrary, at 2.5 g RT, the presence of L-NAME in the perfusing medium increased significantly contractions induced by 10^−6^ M to 10^−3^ M ACh (*P* < .05, Figure 2(b)). At 2.5 g RT, L-NAME increased contractions obtained by 10^−3^ M ACh to 101.08 ± 5.95 g mm^−2^. The pretreatment of preparations with the iNOS inhibitor, SMT or nNOS inhibitor, 7-NI, had no effect on the responsiveness of ASM to ACh (Figure 2(b)).

**Figure 3:**
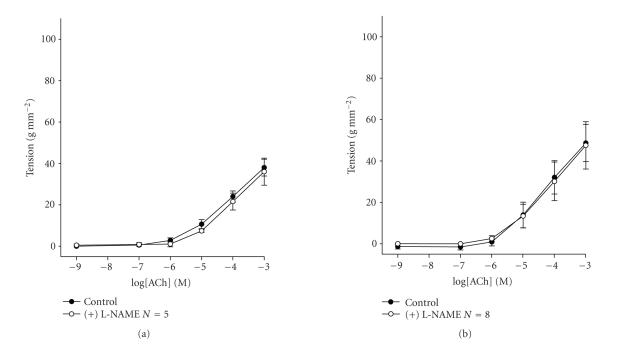
Concentration-effect curves for acetylcholine, performed in Krebs solution with low calcium concentration, at an RT of 0.5 g (a) and 2.5 g (b). Data are means and vertical lines show SE. *N* refers to the number of animals studied.

The decrease in extracellular calcium concentration abolishes the effect of L-NAME on ACh-induced contractions obtained at 2.5 g RT. Thus, at low extracellular calcium concentration, L-NAME had no effect on ACh-induced contractions obtained at either 0.5 g or 2.5 g RT (Figure 3).

**Figure 4:**
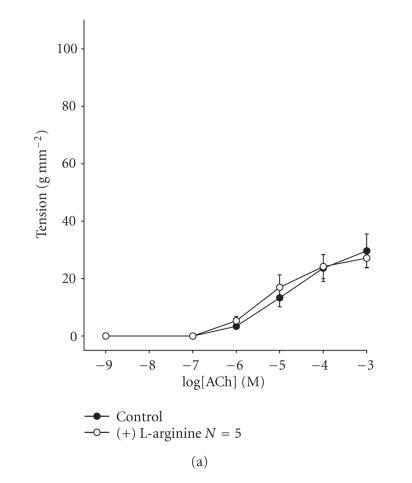

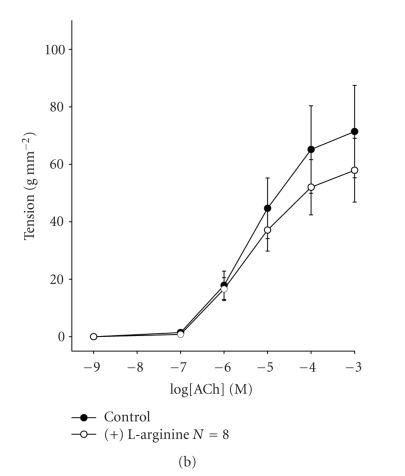
Concentration-effect curves for acetylcholine at an RT of 0.5 g (a) and 2.5 g (b) in the presence of L-arginine. Data are means and vertical lines show SE. *N* refers to the number of animals studied.

**Figure 5:**
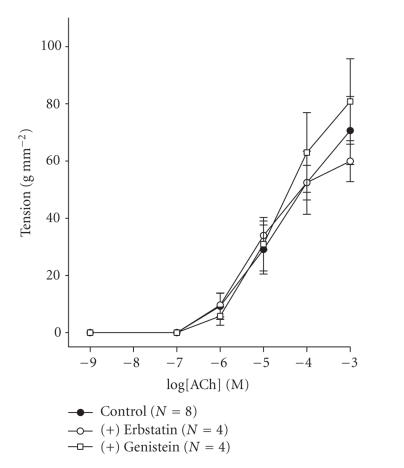
Concentration-effect curves for acetylcholine at an RT of 2.5 g in the presence of tyrosine phosphorylation inhibitors. Data are means and vertical lines show SE. *N* refers to the number of animals studied.

The pretreatment of preparations with the NO precursor, L-arginine, had no effect on ACh-induced contractions in either RT of 0.5 g (Figure 4(a)) or 2.5 g (Figure 4(b)). Similarly, at 2.5 g RT, the presence of the protein tyrosine kinase inhibitor erbstatin A or genistein in the perfusing medium had no effect on ACh-induced contractions (Figure 5).

The alterations of extracellular calcium concentrations as well as the presence of L-NAME or SMT or 7-NI or L-arginine in the perfusing medium had no effect on the passive tension (data not shown).

## 4. DISCUSSION

The alteration of RT from 0.5 g to 2.5 g increased significantly the responsiveness of ASM to acetylcholine. There is evidence that in smooth muscle, mechanical forces may regulate intracellular calcium by multiple pathways [11–16]. The proposed mechanisms are the influx of ions, including calcium, via stretch-activated channels, the membrane depolarization, and the consequent calcium influx via voltage-activated calcium channels, as well as the calcium mobilization from intracellular stores. Although our study was not extended to the underlying mechanism(s) for the RT effect on ASM responsiveness, our results provide evidence for the effect of RT on calcium influx. Thus, results presented in Figure 1 demonstrate that the reduction of extracellular calcium concentration from 2 mM to 0.2 mM abolishes the effect of RT on ASM responsiveness to acetylcholine.

In a previous study [9], we have demonstrated that RT affects endogenous NO release. Consistent with those results, in the present study the nonselective NOS inhibitor L-NAME increased ACh-induced contractions obtained at 2.5 g RT with no effect on ACh-induced contractions obtained at 0.5 g RT. It has been shown by immunohistochemistry [17] and immunoreactivity [18] that rabbit airways express the neuronal and endothelial isoforms of NOS, while the inducible form of the enzyme was essentially absent with the staining for eNOS being the most intense of the three NOS isoenzymes. Considering the above, in the present study we tested the effect of the specific inhibitors of inducible and neuronal NOS, SMT and 7-NI, respectively. As we have shown in Figure 2, at 2.5 g RT, both SMT and 7-NI had no effect on ACh-induced contractions. These results demonstrate that neither iNOS nor nNOS is involved in the increase of NO production at 2.5 g RT.

The eNOS activity could be modulated in a calcium-dependent way that requires an increase of cytosolic calcium concentration and the following promotion of the calmodulin binding to eNOS, and therefore the activity of enzyme [19]. Our results demonstrate that at 2.5 g RT, the decrease of extracellular calcium concentration from 2 mM to 0.2 mM (Figure 3) abolishes the effect of L-NAME on ACh-induced contractions. These results suggest that at 2.5 g RT, the NO production requires normal extracellular calcium concentration. The above results are in consistence with results from a previous study in rabbits, which has demonstrated that extracellular calcium concentration affects the modulatory effect of tracheal epithelium on ACh-induced contractions [10]. Even more there are compelling experimental evidences supporting the existence of voltage-dependent calcium channels on airway epithelial cells that contribute to changes in intracellular calcium concentration observed after mechanical stimulation of the plasma membrane [20–24].

In vessels, it has been demonstrated that L-arginine uptake is involved in NO production caused by mechanical stimuli, but L-arginine is not required for ACh-induced NO production [25, 26] or for the increase in eNOS activity by tyrosine phosphorylation [5–7]. Results from the present study demonstrate that at 2.5 g RT, the extracellular calcium concentration, NOS or tyrosine kinase inhibitors and L-arginine did not alter passive tension. Accordingly, the presence of NO precursor, L-arginine in the perfusing medium did not alter the responsiveness of ASM to ACh at either 0.5 g or 2.5 g RT. Moreover, the tyrosine kinase inhibitors, erbstatin A, and genistein had no effect on ACh-induced contraction obtained at 2.5 g RT, suggesting that in rabbit trachea, tyrosine phosphorylation is not involved in NO production observed at 2.5 g RT. Based on the above, we suggest that RT affects NO production in arrangement with acetylcholine. At 0.5 g RT, the basal level of intracellular calcium is not sufficient to activate eNOS, and thus NO production from epithelial cells. At 2.5 g RT, acetylcholine induces an increase in calcium influx from extracellular space into epithelial cells, which leads to the activation of eNOS and NO production independent of tyrosine phosphorylation and extracellular L-arginine concentration.

Clinical studies have well demonstrated that deep inspiration can act as bronchodilator and bronchoprotector agents. The protective effect of deep inspiration is lost in asthmatics [27–29] and patients with COPD [30]. The reason for the marked difference in the response to deep inspiration between normal and asthmatic subjects is not clear even though several mechanisms have been proposed, including the inhibition of cholinergic tone [31], activation of the inhibitory nonadrenergic, noncholinergic system [32], as well as changes in the organization of the contractile elements of smooth muscle cell [33, 34]. Data available from the present and previous studies of our laboratory [8, 9] suggest that airway epithelium may have an additional modulatory role in this process. As epithelium responds to stretch by modulating eNOS activity, and thus NO production with a consequent reduction of airway responsiveness, this protective mechanism could be impaired in epithelium damage seen in airways diseases in particular asthma [35].
